# Predicting lung cancer stage at diagnosis based on self-reported symptoms and background factors using machine learning models

**DOI:** 10.1038/s41598-026-46710-8

**Published:** 2026-04-08

**Authors:** Tina Gustavell, Noora Sissala, Maria Pernemalm, Haris Babačić, Lars E. Eriksson

**Affiliations:** 1 Department of Neurobiology, Care Sciences and Society, Karolinska Institutet, Stockholm, Sweden; 2 Department of Upper Abdominal Diseases, Theme Cancer, Karolinska University Hospital, Stockholm, Sweden; 3 Science for Life Laboratory, Department of Oncology-Pathology, Karolinska Institutet, Stockholm, Sweden; 4 School of Health and Medical Sciences, City St George’s, University of London, London, UK

**Keywords:** Cancer, Diseases, Health care, Medical research, Oncology, Risk factors

## Abstract

This study aimed to describe and compare background factors and symptoms at diagnosis of patients with non-advanced or advanced stage lung cancer and patients without cancer, and to develop predictive models identifying key variables that contribute to the detection of early and late-stage lung cancer. Univariate logistic regression and three machine learning algorithms were used. Compared to patients without cancer, six background factors and two symptoms differed in non-advanced lung cancer, while 11 background factors and 19 symptoms differed in advanced cases. The machine learning models showed moderate performance in classifying patients with lung cancer from those without cancer. Notably, top predictors extended beyond classic respiratory symptoms. Demographic and lifestyle factors, particularly age, smoking status, and living situation, remained essential alongside symptoms such as pain, appetite loss, weight reduction, and respiratory problems. These findings support integrating clinical, demographic, and patient-reported symptoms to improve lung cancer risk models and refine referral decisions in screening pathways.

**Supplementary Information:**

The online version contains supplementary material available at 10.1038/s41598-026-46710-8.

## Introduction

Lung cancer is the most frequently diagnosed cancer worldwide and the leading cause of cancer-related deaths^1^. One of the reasons for the high mortality is that lung cancer is often diagnosed at a late stage, which reduces the possibility of giving treatments with curative intent. Research indicates that a significant proportion of patients with lung cancer are symptomatic at presentation and that there is a need for multi-symptom assessment as part of the initial evaluation^2^. However, the clinical presentation is rarely specific to lung cancer. Patients may present with symptoms such as cough, chest pain, dyspnoea, hoarseness, weight loss, fatigue, and pain, which can be indicative of various conditions, making early diagnosis crucial but challenging^3–5^.

Patient-reported outcomes can serve as valuable indicators of patient well-being and treatment efficacy, thereby enhancing clinical decision-making^6^. Further, research has shown that specific patient-reported outcomes, such as dizziness, insomnia, and fatigue, correlate significantly with tumour volume changes and overall survival in patients with non-small cell lung cancer undergoing immunotherapy; insomnia has also demonstrated a predictive accuracy of 77% for disease progression^7^. We have previously shown in a cohort referred for investigation of suspected lung cancer that, based on seven background factors and 63 early symptoms, patients’ descriptions of their initial symptoms could aid in predicting lung cancer diagnosis^8^. Further stratification by smoking status enabled the development of models with improved performance, particularly among never-smokers and current-smokers^9^. However, none of these models were stratified by lung cancer stage and, to our knowledge, no studies have incorporated symptom profiles at time of diagnosis into prediction models aimed at identifying different lung cancer stages.

Given the critical importance of detecting lung cancer at an early stage, there is a clear need to further explore how patient-reported outcomes can contribute to the accurate prediction of lung cancer across different stages. A deeper understanding of the relationship between demographic factors, symptoms, and disease stage may not only improve prognostication and inform tailored clinical interventions, but also support patient prioritisation within early detection programs by identifying those at highest risk and enabling timely assessment and management. Therefore, the aim of this study was to describe and compare background factors and symptoms at diagnosis of patients with lung cancer (both those diagnosed at non-advanced and at advanced stages) and patients without cancer, and to develop predictive models identifying key variables that contribute to the detection of early and late-stage lung cancer.

## Methods

### Design

Prospective cohort study.

### Setting and study population

Data were gathered at Karolinska University Hospital where diagnostic workup for suspected lung cancer is centralised in the Stockholm Region; details have been described elsewhere^8^. In brief, all consecutive patients (*n* = 1200) referred to Karolinska University Hospital because of suspected lung cancer were assessed for eligibility. Inclusion criteria were suspected lung cancer and ability to answer a questionnaire in Swedish. Of the 1200 eligible patients, 670 met the inclusion criteria and consented to participate.

### The patient EXperience of bodily changes for lung cancer (PEX-LC) questionnaire

The PEX-LC questionnaire is an e-questionnaire specially developed to support early identification of lung cancer^10^. The e-questionnaire focuses on patients’ own specific pre-diagnostic descriptions of their initial symptoms or sensations, hereafter referred to as symptoms. The questionnaire was developed based on prior qualitative interviews (*n* = 60) conducted at several Swedish pulmonary medicine clinics^10^. The PEX-LC consists of 11 individualised, interactive modules on a touch screen smart tablet: background (e.g., sociodemographic characteristics, comorbidities, and smoking habits), breathing difficulties, cough, phlegm/expectorates, pain/aches/discomfort, fatigue, voice changes, appetite/eating/taste changes, olfactory changes, fever/chills/sweating, and other changes (e.g., general physical condition, malaise, or other emotional changes). There are 342 potential items; 285 that are indicative of the first symptoms the patient noticed that had caused a change in their lives and 57 background variables. The PEX-LC is tailored to allow each individual patient to complete only those items appropriate for the specific individual’s onset of symptoms. In addition to reporting their first symptoms, patients also record if each indicated symptom remains or if any new symptoms have appeared.

### Data collection

Directly before their clinical visit with a pulmonologist, patients who had given written informed consent completed the PEX-LC. Research assistants were available to help when answering the e-questionnaire. An eventual diagnosis of lung cancer within ≤ 12 months from inclusion in the study was retrieved from medical records. Data on the documented stage of lung cancer that patients had been diagnosed with within three months of completing the questionnaire were used to determine stage of disease at the time of symptom report. Those with a documented staging outside of this timeframe were excluded from the present analysis. Stage I-II was categorised as non-advanced stage lung cancer. Locally advanced stage IIIa was also grouped with the non-advanced stage due to the possibility of surgical treatment with curative intent for patients with this stage. Stage IIIb-IV was categorised as advanced stage.

### Data analysis

#### Data selection

**Fig. 1:**
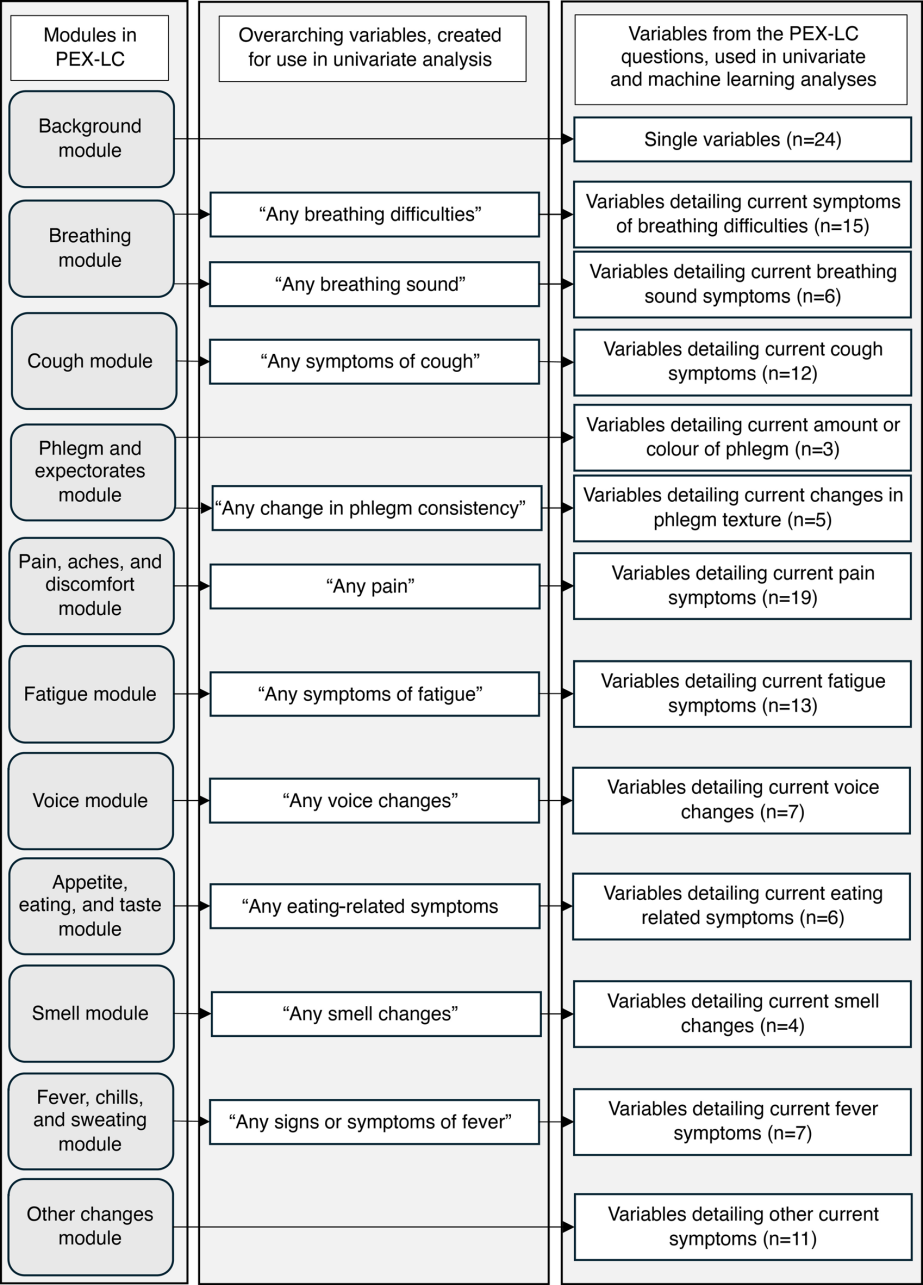
Summary of background factors and symptom variables included in the PEX-LC questionnaire. Modules included in the PEX-LC questionnaire, overarching variables for each module, and number of variables (reflecting current symptoms) in each module are shown.

Of the 670 patients who consented to participate, 506 had complete data from the PEX-LC questionnaire^8^. For the aim of the present study, patients diagnosed with lung cancer but for whom information about stage at diagnosis was missing (n = 16) were removed, leaving a sample of 486 patients, with complete data for all variables. In our previous analysis, we focused on variables representing initial symptoms. In the present analysis, we were interested in symptoms reported at time of diagnosis and their association with lung cancer staging. To investigate this, new variables were created to reflect symptoms present at diagnosis. These were based on whether an initial symptom had persisted or if a new symptom had emerged later in the trajectory prior to diagnosis, resulting in variables that capture the patient’s current symptom profile. Further, all symptoms in a module (except for ’background’ and ’other changes’) were merged to create one or more overarching variables for that module. Based on the nature of the dataset, this gave 142 potential items: 118 symptoms (including ten overarching variables) and 24 background variables (Fig. 1).

#### Univariable analysis

To describe background factors and symptoms, frequencies, percentage, means, and standard deviations (SD) were calculated separately for the three groups: patients without cancer, patients with non-advanced stage lung cancer, and patients with advanced stage lung cancer. Categorical data are presented as frequencies (n) and percentages (%), while continuous data are given as means and SD. To compare patients without cancer with patients with non-advanced stage and advanced stage, respectively, separate analyses were performed, one comparing non-advanced stage lung cancer to no cancer and one comparing advanced stage cancer to no cancer. Two-sided Wald tests for differences between the groups were performed using binary logistic regression. Statistical significance was set at *p* < 0.05.

#### Machine learning

##### Model training and performance evaluation

To explore combinations of background factors and symptoms predictive of lung cancer stage, and to account for potential interactions and non-linear relationships between predictors, supervised machine learning models were trained to classify patients with non-advanced stage lung cancer versus no cancer (non-advanced stage models, *n* = 342 patients) and patients with advanced stage lung cancer versus no cancer (advanced stage models, *n* = 336 patients). To capture both linear and non-linear effects, regularised logistic regression (RLR) and tree-based ensemble models, including random forest (RF) and extreme gradient boosting (XGB), were applied.

Of the initial 142 variables, ten overarching symptom variables, two non-specified variables named (‘other problems’), and one background variable, (’other comorbidities’), were excluded from analysis, leaving 129 variables available for modelling. The overarching variables were excluded because they represented a sum of other variables used for training, which could distort interpretation by introducing dismissal or increasing the importance of already included predictors. The ‘other problems’ and ‘other comorbidities’ variables were excluded since their vague definitions complicated their interpretability and reliability in a predictive model.

Model performance was estimated using 10-fold cross-validation, a method that has been empirically shown to provide test error rate estimates that balance bias and variance effectively^11^. The cross-validation was repeated 10 times with different random data splits to ensure robust estimates. Models were trained to maximize area under the curve (AUC). Age was standardised within each cross-validation fold. Hyperparameter tuning was performed alongside model training using grid search. Model performance and variable importance were reported for models trained with the hyperparameter combination yielding the highest mean AUC over all folds and repeats.

In addition to AUC, balanced accuracy (mean of sensitivity and specificity), sensitivity (true positive rate), specificity (true negative rate), positive predictive values (PPV), and negative predictive values (NPV) were reported at both the default probability cutoff of 0.5, as well as adjusted probability cutoffs determined by the maximum Youden’s index in each fold. All performance metrics were reported as the mean with 95% confidence intervals over folds and repeats (*n* = 100). To facilitate interpretation of model performance, commonly used thresholds from predictive modelling in clinical cancer research were applied. All metrics were interpreted using the following thresholds: low: < 0.60, moderate: 0.60–0.80, and high: > 0.80.

To evaluate model performance using background factors or symptoms only, additional analyses were conducted using the same classification method and performance metrics. Models were trained on the same cross validation folds as in the main analysis to ensure comparability.

To evaluate how well predicted probabilities reflected actual lung cancer risk, we assessed model calibration in the hold-out validation sets of each cross-validation fold. Calibration curves were plotted using LOESS smoothing, while calibration intercepts and slopes were obtained from a logistic calibration model, that is, logistic regression of observed outcomes on the logit of predicted probabilities. Decision curve analysis was performed to evaluate the potential clinical utility of the models, by calculating and visualizing net benefit across probability thresholds, comparing the trained models to strategies of “treat all” or “treat none”.

##### Variable importance

To assess the contribution of each predictor to the models’ decision-making processes, model-internal estimates of variable importance were extracted for each cross-validation fold using the varImp function from the caret R package (‘model-based importance’). For RLR, the function reports the importance as the absolute value of the regression coefficient; for RF, it reports tree-level permutation importance based on out-of-bag samples, calculated as the average decrease in classification accuracy upon permutation of the variable; and for XGB, it reports gain importance, which is calculated as the average improvement in the models predictions when the variable is used to split a node in a tree.

In addition, the overall influence of each predictor on model performance was assessed using a model-agnostic permutation approach (‘permutation-based importance’). In this approach, after model training, the values for a single variable are randomly shuffled in the held-out validation data and the AUC is re-estimated to calculate the AUC change related to that variable. A drop in the AUC therefore gives a positive variable importance.

For both methods, the mean importance and the frequency of occurrence among the top ten variables (conditional on importance > 0) was calculated across all folds and repeats. Mean importance values were scaled using min-max scaling to a range of 0–1 for visual presentation of the values, for each algorithm separately. Variable selection frequency was calculated based on the model-based importance (frequency of importance > 0 across folds). Based on permutation-based importance, the frequency of positive contribution to the AUC was reported. As a result, the importance of each predictor was assessed using six different metrics, the median rank across these metrics was used to identify the top predictors.

##### Software

The univariable analysis was conducted using R version 4.2.1 with packages broom and dplyr. The machine learning analysis was performed using R version 4.4.2 and the following R packages: caret, recipes, glmnet, ranger, xgBoost, and pROC. Permutation importance was calculated with the vi function in the vip R package. Calibration assessment was performed using the CalibrationCurves package, and decision curve analysis was performed using the rmda package.

### Ethical considerations

This study was carried out according to the Declaration of Helsinki^12^. Data were pseudonymised to protect the privacy of the study participants, meaning that only researchers directly involved in the project had access to personal identity data. Ethical approval was obtained from the Regional Ethical Review Board in Stockholm, Sweden (Reg.no 2014/1290–32). All participants were informed about the study both verbally and in writing, and all provided written consent.

## Results

Of the 486 patients, 192 (39.5%) were not diagnosed with cancer and 294 (60.5%) were diagnosed with lung cancer. Of the latter, 150 (51.0%) were diagnosed with non-advanced stage (I-IIIa) and 144 (49.0%) with advanced stage (IIIb-IV) lung cancer.

### Background factors indicative of lung cancer

Description and comparison of background factors for patients without cancer and those with non-advanced or advanced stage lung cancer are presented in Table 1. Irrespective of stage, the patients diagnosed with lung cancer were older (*p* < 0.001), more often living alone (*p* < 0.001), and more often current daily smokers (*p* < 0.001 for non-advanced stage and *p* = 0.001 for advanced stage). Of those who were daily smokers at the time of the investigation, patients with cancer were more likely to have reduced their smoking in the past two years as compared to patients not diagnosed with cancer (*p* = 0.045 for non-advanced stage and *p* = 0.009 for advanced stage).

**Table 1:** Descriptions and comparisons of background factors reported by patients without cancer (*n* = 192), with non-advanced stage lung cancer (*n* = 150) and with advanced stage lung cancer (*n* = 144).

Background data	Total (*n* = 486)	No cancer (*n* = 192)	Non-advanced stage (*n* = 150)					Advanced stage (*n* = 144)				
	Desc.^a^	Desc.^a^	Desc.^a^	OR^b^	95% CI^b^		*P* ^b^	Desc.^a^	OR^c^	95% CI^c^		*P* ^c^
Age in years, mean (SD)	68 (10.8)	64.5 (12.7)	70.8 (8.6)	1.06	1.03	1.08	**< 0.001**	70.2 (8.6)	1.05	1.03	1.07	**< 0.001**
Male, n (%)	247 (50.82)	111 (57.81)	71 (47.33)	0.66	0.43	1.01	0.054	65 (45.14)	0.60	0.39	0.93	**0.022**
Living alone, n (%)	177 (36.42)	47 (24.48)	65 (43.33)	2.36	1.49	3.76	**< 0.001**	65 (45.14)	2.54	1.60	4.06	**< 0.001**
University education, n (%)	176 (36.21)	78 (40.62)	45 (30.0)	0.63	0.40	0.98	**0.043**	53 (36.81)	0.85	0.54	1.33	0.478
Born in Sweden, n (%)	404 (83.13)	158 (82.29)	124 (82.67)	1.03	0.59	1.81	0.928	122 (84.72)	1.19	0.67	2.17	0.554
Cold, flu, pneumonia past two years, n (%)	338 (69.55)	146 (76.04)	96 (64)	0.56	0.35	0.90	**0.016**	96 (66.67)	0.63	0.39	1.02	0.059
Antibiotic use due to airway problems past two years, n (%)	186 (38.27)	88 (45.83)	57 (38)	0.72	0.47	1.12	0.146	41 (28.47)	0.47	0.30	0.74	**0.001**
Asthma, n (%)	66 (13.58)	34 (17.71)	18 (12)	0.63	0.34	1.16	0.147	14 (9.72)	0.50	0.25	0.95	**0.041**
Emphysema, n (%)	22 (4.53)	6 (3.12)	11 (7.33)	2.45	0.91	7.27	0.084	5 (3.47)	1.12	0.32	3.77	0.860
Asbestos-related disease, n (%)	3 (0.62)	1 (0.52)	1 (0.67)	1.28	0.05	32.60	0.861	1 (0.69)	1.34	0.05	33.97	0.838
Chronic bronchitis, n (%)	10 (2.06)	5 (2.6)	3 (2)	0.76	0.15	3.16	0.715	2 (1.39)	0.53	0.07	2.48	0.448
COPD, n (%)	90 (18.52)	25 (13.02)	31 (20.67)	1.74	0.98	3.12	0.060	34 (23.61)	2.06	1.17	3.68	**0.013**
Pleural fluid, n (%)	50 (10.29)	24 (12.5)	12 (8)	0.61	0.28	1.24	0.182	14 (9.72)	0.75	0.37	1.50	0.427
Anaemia, n (%)	7 (1.44)	3 (1.56)	1 (0.67)	0.42	0.02	3.34	0.458	3 (2.08)	1.34	0.24	7.34	0.722
Heart disease, n (%)	60 (12.35)	24 (12.5)	22 (14.67)	1.20	0.64	2.25	0.560	14 (9.72)	0.75	0.37	1.50	0.427
Angina pectoris, n (%)	19 (3.91)	6 (3.12)	3 (2)	0.63	0.13	2.44	0.522	10 (6.94)	2.31	0.84	6.94	0.113
Pneumonia, n (%)	121 (24.90)	57 (29.69)	38 (25.33)	0.80	0.49	1.30	0.373	26 (18.06)	0.52	0.30	0.87	**0.015**
Other comorbidities, n (%)	70 (14.40)	26 (13.54)	27 (18)	1.40	0.78	2.53	0.260	17 (11.81)	0.85	0.44	1.63	0.638
No comorbidities, n (%)	148 (30.45)	57 (29.69)	44 (29.33)	0.98	0.61	1.57	0.943	47 (32.64)	1.15	0.72	1.83	0.563
Weight reduction past year, n (%)	194 (39.92)	65 (33.85)	52 (34.67)	1.04	0.66	1.62	0.875	77 (53.47)	2.25	1.44	3.51	**< 0.001**
Weight increase past year, n (%)	62 (12.76)	32 (16.67)	22 (14.67)	0.86	0.47	1.54	0.615	8 (5.56)	0.29	0.12	0.63	**0.003**
Current daily smoker (incl. quitters past year), n (%)	145 (29.84)	37 (19.27)	56 (37.33)	2.50	1.54	4.09	**< 0.001**	52 (36.11)	2.37	1.45	3.90	**0.001**
Increased smoking past two years, n (%)	7 (1.44)	2 (1.04)	3 (2)	1.94	0.32	14.86	0.472	2 (1.39)	1.34	0.16	11.26	0.772
Reduced smoking past two years, n (%)	56 (11.52)	13 (6.77)	20 (13.33)	2.12	1.03	4.51	**0.045**	23 (15.97)	2.62	1.29	5.51	**0.009**

^a^Desc. = Descriptive values of mean (SD), or n (%).

^b^Odds ratio (OR), upper and lower values for 95% confidence intervals (CI), and *p*-value for univariate analysis using logistic regression comparing patients without cancer to patients with non-advanced stage lung cancer.

^c^Odds ratio (OR), upper and lower values for 95% confidence intervals (CI), and *p*-value for univariate analysis using logistic regression comparing patients without cancer to patients with advanced stage lung cancer.

Significant values are in bold.

Patients with non-advanced stage lung cancer were less likely to have a university education (*p* = 0.043) or have had a cold, flu, or pneumonia in the past two years (*p* = 0.016) compared to patients without cancer.

Patients with advanced stage lung cancer were more likely to be men (*p* = 0.022), to have been treated with antibiotics for airway problems (*p* = 0.001), and to have been diagnosed with asthma (*p* = 0.041), COPD (*p* = 0.013), or pneumonia (*p* = 0.015) compared to patients without cancer. Further, they were more likely to have experienced weight loss (*p* < 0.001) and less likely to have experienced weight gain (*p* = 0.003) compared to patients with no cancer.

### Symptoms indicative of lung cancer

Comparisons of symptoms at diagnosis between patients with non-advanced stage lung cancer and patients without cancer showed an overall similar symptom experience. Exceptions were experiencing a whistling breathing (*p* = 0.017), which was more common amongst patients later diagnosed with non-advanced stage lung cancer, and having experienced fever (*p* = 0.015), which was more common amongst patients not diagnosed with lung cancer. Statistically significant differences in the univariable analysis are shown in Table 2. All results from the univariable symptom analysis are presented in Supplementary Table 1.

**Table 2:** Descriptions and comparisons of symptoms with statistically significant differences reported by patients without cancer (*n* = 192), with non-advanced stage lung cancer (*n* = 150) and with advanced stage lung cancer (*n* = 144).

Symptom / sensation	Total (*n* = 486)	No Cancer (*n* = 192)	Non-advanced stage (*n* = 150)					Advanced stage (*n* = 144)				
	*n* (%)	*n* (%)	*n* (%)	OR^a^	95% CI^a^		*P* ^a^	*n* (%)	OR^b^	95% CI^b^		*P* ^b^
Any breathing difficulty	246 (50.62)	87 (45.31)	74 (49.33)	1.18	0.77	1.80	0.460	85 (59.03)	1.74	1.13	2.70	**0.013**
Hard to get air	81 (16.67)	29 (15.1)	15 (10)	0.62	0.31	1.20	0.165	37 (25.69)	1.94	1.13	3.37	**0.017**
Hard to catch breath	28 (5.76)	8 (4.17)	6 (4)	0.96	0.31	2.82	0.939	14 (9.72)	2.48	1.03	6.36	**0.048**
Gasping for air	22 (4.53)	3 (1.56)	8 (5.33)	3.55	1.01	16.42	0.065	11 (7.64)	5.21	1.59	23.36	**0.013**
Tightness in throat	39 (8.02)	8 (4.17)	12 (8)	2.00	0.81	5.23	0.140	19 (13.19)	3.50	1.53	8.71	**0.004**
Breathing sound: rattly/wheezing	47 (9.67)	15 (7.81)	10 (6.67)	0.84	0.36	1.91	0.687	22 (15.28)	2.13	1.07	4.34	**0.033**
Breathing sound: whistling	24 (4.94)	4 (2.08)	12 (8)	4.09	1.39	14.86	**0.017**	8 (5.56)	2.76	0.85	10.53	0.102
Irritating cough	74 (15.23)	20 (10.42)	26 (17.33)	1.80	0.97	3.41	0.065	28 (19.44)	2.08	1.12	3.90	**0.021**
Any pain	211 (43.42)	70 (36.5)	63 (42.0)	1.26	0.81	1.96	0.297	78 (54.17)	2.06	1.33	3.21	**0.001**
Persistent pain	45 (9.26)	13 (6.77)	10 (6.67)	0.98	0.41	2.30	0.970	22 (15.28)	2.48	1.22	5.24	**0.014**
Pain comes and goes	93 (19.14)	31 (16.15)	22 (14.67)	0.89	0.49	1.61	0.708	40 (27.78)	2.00	1.18	3.41	**0.011**
Back pain	52 (10.70)	13 (6.77)	13 (8.67)	1.31	0.58	2.93	0.513	26 (18.06)	3.03	1.52	6.32	**0.002**
Any symptoms of fatigue	246 (50.62)	105 (54.69)	82 (54.67)	1.00	0.65	1.54	0.997	99 (68.75)	1.82	1.16	2.88	**0.009**
Less energy to do things	151 (31.07)	55 (28.65)	42 (28)	0.97	0.60	1.55	0.895	61 (42.36)	1.83	1.16	2.89	**0.009**
Weakness in the legs	37 (7.61)	9 (4.69)	14 (9.33)	2.09	0.89	5.16	0.095	15 (10.42)	2.36	1.02	5.78	**0.049**
Rougher voice	42 (8.64)	12 (6.25)	10 (6.67)	1.07	0.44	2.55	0.876	19 (13.19)	2.28	1.08	4.99	**0.033**
Any eating changes	139 (28.60)	45 (23.44)	42 (28)	1.27	0.78	2.07	0.337	65 (45.14)	2.69	1.69	4.31	**< 0.001**
Loss of appetite	97 (19.96)	26 (13.54)	29 (19.33)	1.53	0.86	2.74	0.150	48 (33.33)	3.19	1.88	5.54	**< 0.001**
Early satiety	57 (11.73)	20 (10.42)	18 (12)	1.17	0.59	2.31	0.644	36 (25)	2.87	1.59	5.29	**0.001**
Feeling chilly	27 (5.56)	9 (4.69)	12 (8)	1.77	0.73	4.44	0.211	17 (11.81)	2.72	1.20	6.57	**0.019**
Fever	32 (6.58)	15 (7.81)	1 (0.67)	0.08	0.00	0.40	**0.015**	12 (8.33)	1.07	0.48	2.36	0.862

^a^Odds ratio (OR), upper and lower values for 95% confidence intervals (CI), and *p*-value for univariate analysis using logistic regression comparing patients without cancer to patients with non-advanced stage lung cancer.

^b^Odds ratio (OR), upper and lower values for 95% confidence intervals (CI), and *p*-value for univariate analysis using logistic regression comparing patients without cancer to patients with advanced stage lung cancer.

Significant values are in bold.

Statistically significant differences in several symptoms could be observed for patients with advanced stage lung cancer compared to patients not diagnosed with cancer. Regarding breathing-related symptoms, any breathing difficulty (*p* = 0.013), hard to get air (*p* = 0.017), hard to catch breath (*p* = 0.048), gasping for air (*p* = 0.013), tightness in the throat (*p* = 0.004), a rattly/wheezing sound when breathing (*p* = 0.033), and an irritating cough (*p* = 0.021) were more common amongst patients with advanced stage lung cancer. Regarding pain, reporting any pain (*p* = 0.001), persistent pain (*p* = 0.014), pain that comes and goes (*p* = 0.011), and back pain (*p* = 0.002) were more common amongst patients with advanced stage lung cancer. Further, reporting any symptoms of fatigue (*p* = 0.009), having less energy to do things (*p* = 0.009), weakness in the legs (*p* = 0.049), having a rougher voice (*p* = 0.033), and feeling chilly (*p* = 0.019) were more common amongst patients with advanced stage lung cancer. Regarding eating-related symptoms, experiencing any eating problems (*p* < 0.001), loss of appetite (*p* < 0.001), and early satiety (*p* = 0.001) were more common amongst patients with advanced stage lung cancer. Statistically significant differences in the univariable analysis are shown in Table 2.

### Combining background factors and symptoms for lung cancer prediction using machine learning

To explore combinations of background factors and symptoms predictive of lung cancer stage, we trained machine learning models to distinguish non-advanced stage from no cancer (non-advanced stage models) and advanced stage from no cancer (advanced stage models). We applied three different algorithms, RLR, RF, and XGB, to accommodate both linear and non-linear effects, and to assess the consistency of variable selection and importance across models.

**Fig. 2:**
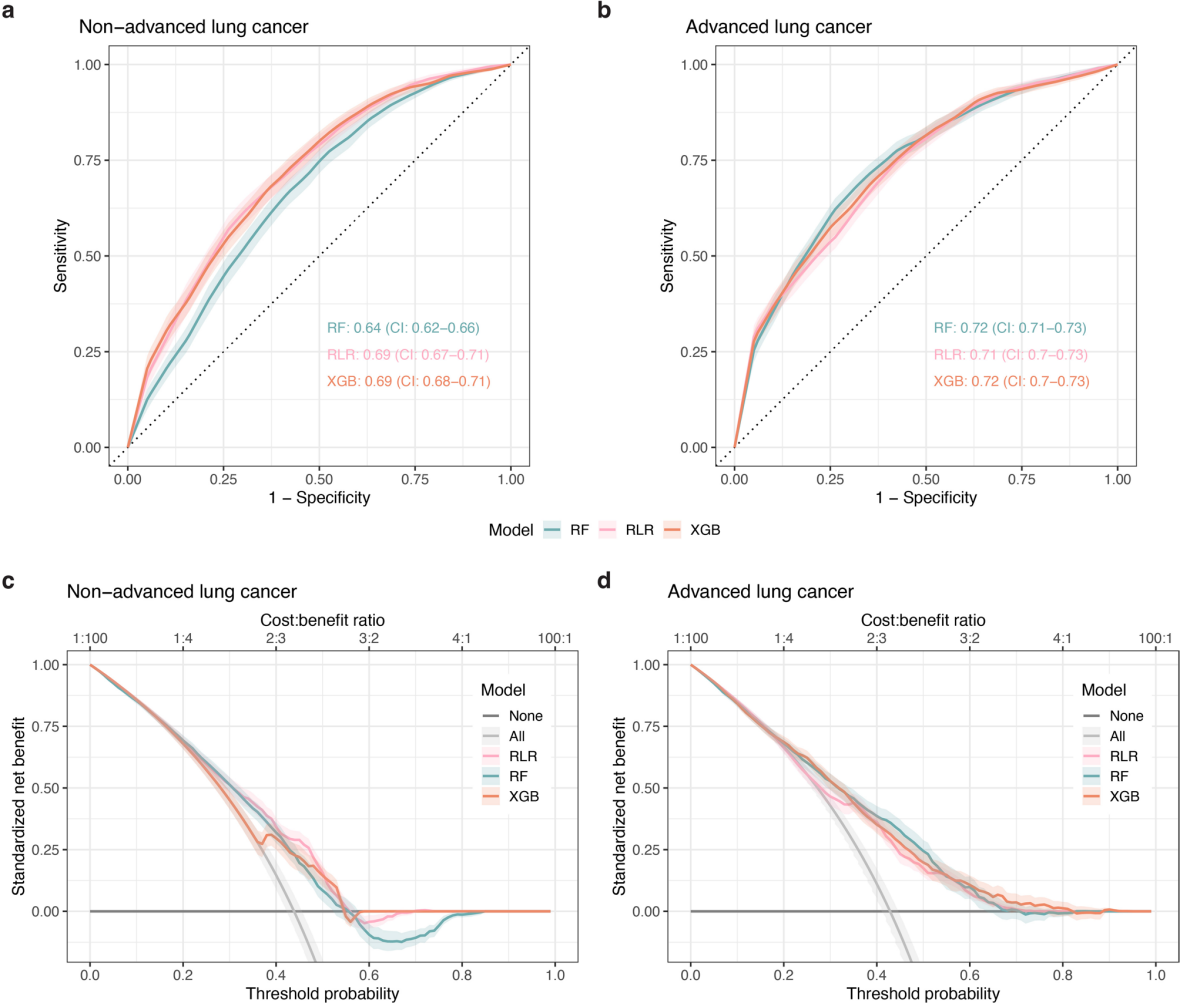
Machine learning model performance. (**a,b**) Mean ROC curves across cross-validation folds (*n* = 100) for regulari**s**ed logistic regression (RLR), random forest (RF), and extreme gradient boosting (XGB) models predicting either (**a**) non-advanced or (**b**) advanced stage lung cancer. The curves were calculated using the vertical averaging method. The mean area under the curve (AUC) with 95% confidence intervals (CI) for each model is annotated on the plots. (**c,d**) Decision curve analysis for (**c**) non-advanced stage and (**d**) advanced-stage models. Decision threshold probabilities are plotted against the observed standardised net benefit, with the corresponding cost-to-benefit ratio shown above the plot. Curves for referring ‘None’ or ‘All’ patients are shown for reference.

The models demonstrated limited ability in distinguishing between lung cancer and no cancer, with average AUCs ranging from 0.64 to 0.69 for the non-advanced stage models, and from 0.71 to 0.72 for the advanced stage models, depending on the algorithm used for training (Fig. 2a and b, Supplementary Table 2). Although the models showed slightly better performance in distinguishing patients with advanced stage cancer than patients with non-advanced stage cancer from patients without cancer, the difference was minor and not statistically significant.

While RLR, RF, and XGB models performed similarly in terms of AUC and balanced accuracy in both analyses, they differed in their sensitivity-specificity trade-offs (Supplementary Fig. 1a, Supplementary Table 3). Nonetheless, the sensitivity was on average lower than the specificity for all algorithms, indicating difficulty in correctly identifying lung cancer cases, particularly for the non-advanced stage models. Calibration assessment revealed that all non-advanced stage models, as well as the advanced stage RLR models, were poorly calibrated and tended to misestimate lung cancer likelihood (Supplementary Fig. 2). Moreover, the narrow range of predicted probabilities suggested that the models were underfit. In contrast, the remaining advanced stage models demonstrated good calibration, with predicted probabilities aligned with observed cancer frequencies.

To assess the potential clinical benefit of the models, we performed a decision curve analysis, which estimates the net benefit and cost-to-benefit ratio of using a model to guide clinical decisions across probability thresholds (Fig. 2c and d). The analysis suggested that at thresholds of approximately 0.38–0.55 for non-advanced stage and 0.30–0.65 for advanced stage, the models may provide clinical benefit for stratifying patients for further lung cancer evaluation, as indicated by a higher net benefit compared to strategies of referring all or no patients. However, sensitivity at the default probability threshold of 0.5 was suboptimal for an early detection setting. Therefore, we calculated Youden’s index for different thresholds, to explore the sensitivity-specificity trade-off for each of them. Thresholds with highest Youden’s index were consistently in the range of 0.40–0.47 (Supplementary Table 4). At these lower thresholds, models favoured sensitivity over specificity and NPV over PPV (Supplementary Fig. 1b, Supplementary Table 3), demonstrating the relevance of exploring how probability thresholds affect the clinical utility of the models. To further assess the individual contribution of background factors and symptoms to prediction of lung cancer stage, we trained separate models based only on background variables or only on symptoms. Overall, regardless of algorithm, models trained on only background factors had higher discriminative performance than models trained on only symptoms, and comparable performance to the main models that were trained on both variable types (Supplementary Fig. 3). This was substantially evident for the non-advanced stage models, where symptoms-trained models were no better than chance (Supplementary Fig. 3).

### Identifying key variables for lung cancer prediction through machine learning

**Fig. 3:**
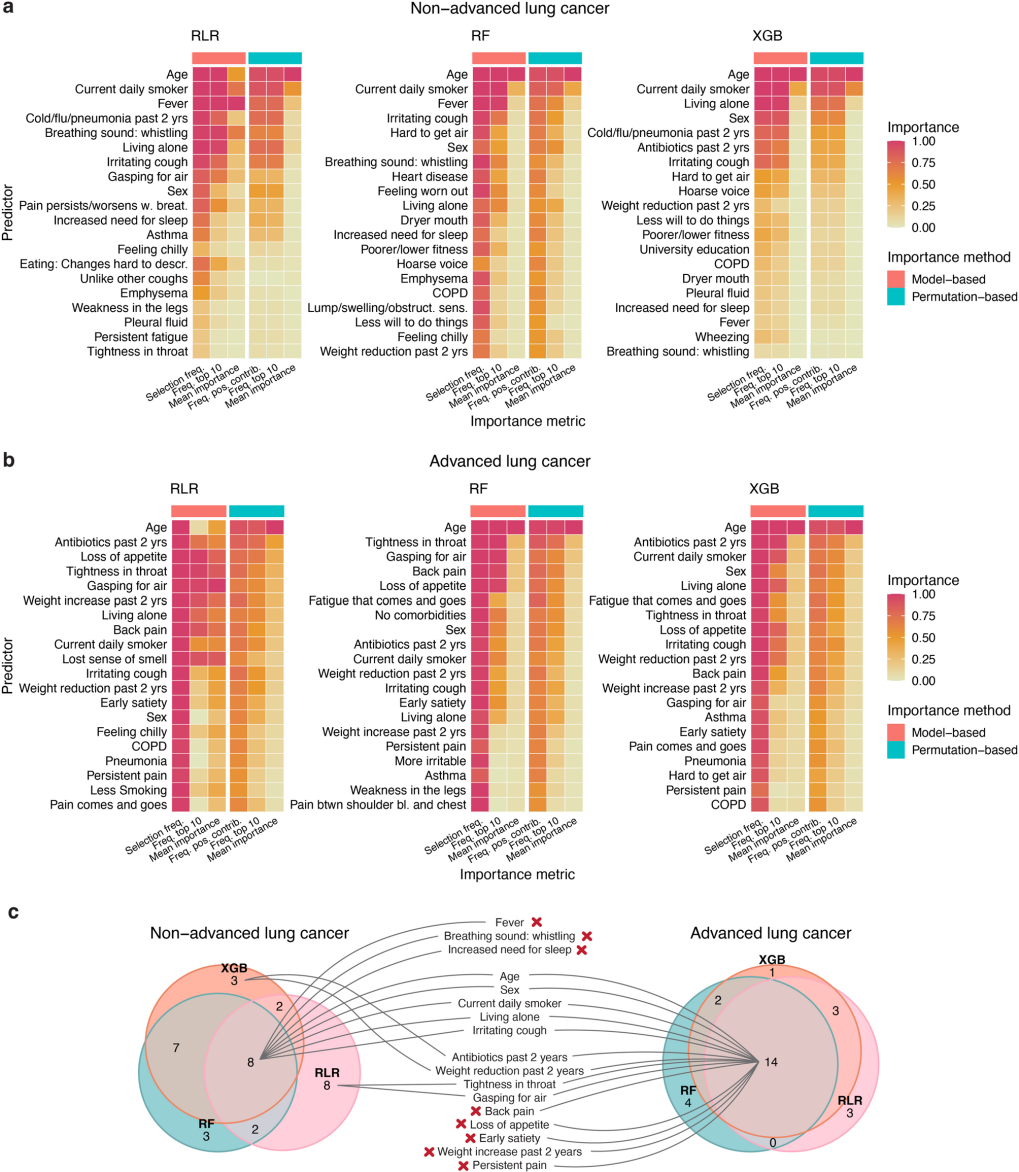
Top lung cancer predictors identified in machine learning analyses. (**a,b)** Summary of model- and permutation-based variable importance metrics for top 20 most important variables of regulari**s**ed logistic regression (RLR), random forest (RF), and extreme gradient boosting (XGB) models predicting (**a**) non-advanced stage and (**b**) advanced stage lung cancer. Top variables were selected based on the median rank over all importance metrics. Selection frequency: how often a variable had a model-based importance > 0 across cross validation folds (*n* = 100). Freq. top 10: frequency of appearing in the top 10 most important variables across folds. Freq. pos. contrib.: frequency of positive contribution to model performance (area under the curve, AUC). Mean importance: scaled mean importance over folds. (**c**) Venn diagrams showing the intersections between top-ranked variables of each algorithm. In the centre, variables consistently ranked high by all three algorithms are compared between non-advanced stage and advanced stage models. Red crosses indicate variables that were deemed highly important only in either non-advanced or advanced stage models.

We evaluated the contribution of background factors and symptoms to model predictions and performance using both model-based and permutation-based metrics of variable importance (see Methods). The most important variables were identified across folds based on the median rank using both model-based metrics, which included selection frequency, frequency of occurrence in the top ten, and mean importance, as well as permutation-based metrics, which included frequency of positive contribution to model performance, frequency among the top ten with positive contribution, and mean importance (Fig. 3a and b).

Several variables had consistently high importance across all metrics and algorithms. The background factors age, sex, smoking status, and living alone, along with the symptom irritating cough appeared among the top 20 variables ranking for all models, regardless of cancer stage. For the non-advanced stage models, three additional variables appeared in the top 20 ranking for all three algorithms, namely symptoms fever, whistling breathing, irritating cough, and increased need for sleep. For the advanced stage models, nine additional variables were in the top 20 ranking for all three algorithms, namely the background factor antibiotic use in past two years, and symptoms loss of appetite, early satiety, weight reduction in past two years, weight increase in past two years, tightness in throat, gasping for air, back pain, and persistent pain. Of these, persistent pain, back pain, loss of appetite, early satiety, and weight increase in past two years were not among the top variables of any of the non-advanced stage models. Taken together, these results indicate that the background factors identified as important by the models were more consistent across lung cancer stages, whereas symptom variables varied more in their importance between non-advanced and advanced stage models. In addition, fewer variables achieved consistently high importance across folds (Supplementary Figs. 4–5) and algorithms (Fig. 3c) for the non-advanced stage models. These observations likely explain why models trained only on background factors performed better than models based only on symptoms.

Age had a consistently large impact on model performance (AUC) across folds and algorithms (Supplementary Fig. 4), with variable permutation incurring an average AUC loss of 0.10–0.12 for non-advanced stage models, and 0.04–0.09 for advanced stage models. For non-advanced stage models, being a current smoker also had a relatively large impact on models’ AUC (0.04–0.07), but apart from these variables, the average individual effect on AUC of most variables was small. This suggests that a combination of several background factors and/or symptoms was needed to accurately distinguish between patients with lung cancer and patients with no cancer. Indeed, on average, non-advanced stage models used 12–55 variables for prediction, while advanced stage models used 37–64 variables (Supplementary Fig. 6). Apart from background factors, several symptoms had a positive contribution to model AUC and were often deemed highly important by the models (Fig. 3a and b, Supplementary Figs. 4–5). For non-advanced cancer, the most notable included fever, whistling breathing, irritating cough, gasping for air, and a feeling that it was hard to get air. For advanced cancer, these included, among others, a feeling of tightness in the throat, gasping for air, back pain, loss of appetite, and fatigue that came and went.

## Discussion

This study aimed to identify key patient background factors and patient-reported symptoms that can support early diagnosis of lung cancer. Significant differences were observed between individuals who were subsequently diagnosed with lung cancer, particularly at advanced stages, and those not receiving a cancer diagnosis. Demographic factors, such as older age and being a current daily smoker, were more prevalent among patients with cancer, aligning with previous studies that emphasise these as key risk factors^13,14^. In addition, living alone and recent weight loss also ranked highly, reflecting earlier findings on the influence of social isolation and physical deterioration in cancer trajectories^15^. These variables appeared among the top predictors, suggesting that combining demographic and clinical information adds important predictive value beyond symptoms alone. While these factors do not serve as definitive criteria for referral for investigation, their inclusion in risk assessment tools can enhance early identification of high-risk individuals, potentially even before noticeable symptoms emerge. This also highlights the need to support individuals with lower socioeconomic status and those living alone in accessing timely medical care.

In univariable analyses of symptoms, only experiencing a whistling sound when breathing and not having fever separated patients with non-advanced stage lung cancer from those without cancer. These findings reflect the diagnostic challenge of early-stage lung cancer, where symptoms are often subtle or non-specific, a pattern similarly noted in a prior study^16^. In contrast, the multivariable machine learning analysis identified several additional symptoms as important predictors of non-advanced lung cancer, including hoarse voice, difficulty getting air, and a need to gasp for air, symptoms previously recognised as strong indicators of lung cancer risk^16^. The fact that the machine learning revealed these additional predictors demonstrates its utility in uncovering complex symptom patterns. These findings emphasise the importance of not dismissing vague or isolated symptoms in clinical encounters, such as hoarseness, particularly when multiple symptoms co-occur. Improving recognition of these early symptom patterns, both in clinical risk assessment tools and among healthcare professionals, is key to enhancing early-stage detection, when curative treatment is still possible.

The patients with advanced stage lung cancer reported a broader range of differentiating symptoms, including dyspnoea, weight loss, fatigue, and gastrointestinal issues such as early satiety and loss of appetite, all previously associated with cancer progression and reduced quality of life^16,17^. Pain and systemic symptoms such as chills were also more common in this group compared to patients without cancer, reflecting the increased symptom burden of advanced disease previously reported^17,18^. These findings align with prior studies showing that patients with advanced lung cancer often present with more pronounced and debilitating symptoms and underscore the need for greater public awareness to encourage earlier consultation for concerning symptoms, as well as improved symptom recognition within primary care to support timely diagnosis^18,19^. In comparison with our previous models^8,9^ which evaluated occurring symptoms months or years before diagnosis, the present study, focusing on current symptoms in proximity to diagnosis, identified overlapping top symptom predictors, namely whistling breathing, irritating cough, tightness in throat, gasping for air, back pain, loss of appetite, early satiety, and persistent pain.

While many breathing-related symptoms were common across all patients, several, such as gasping for air, irritating cough, and whistling breathing, ranked highly in the models for non-advanced stage lung cancer. In contrast, symptoms other than respiratory were rarely among the top-ranked predictors in these models. In the advanced stage models, both respiratory symptoms and non-specific cancer symptoms, including appetite loss, showed high importance. This suggests that, apart from key breathing-related symptoms, non-specific symptoms also contribute meaningfully to prediction. The clinical relevance of these non-specific symptoms is supported by previous research showing high prevalence of appetite loss among patients with lung cancer, especially for advanced stages^20,21^. These findings highlight the importance of considering both respiratory and non-specific symptoms in clinical assessment.

The predictive models developed in this study demonstrated modest performance in both non-advanced and advanced stage lung cancer. At a chance-defined probability cutoff of 0.5, sensitivity was low, which would be suboptimal in an early detection setting and result in missed cases, particularly in non-advanced stages. However, lowering probability thresholds can shift model performance towards higher sensitivity and NPV, albeit at the expense of specificity and PPV. While our results suggest that symptoms and background information might be insufficient on their own for reliable screening or diagnosis, they can still be useful in supporting prioritisation of patients for diagnostic evaluation. Although screening with low dose computed tomography reduces lung cancer mortality, its effectiveness depends on the appropriate selection of high-risk individuals to minimise unnecessary radiation exposure and anxiety, and challenges related to overdiagnosis and false positives remain^22^. Since most current screening strategies focus primarily on traditional risk factors, such as age and smoking, further inclusion of symptom data and other background information might help refine screening and support earlier lung cancer detection. This is especially relevant for individuals who do not yet meet imaging thresholds based on age or smoking history alone. Future work could explore combining background and symptom data with molecular modalities, particularly minimally invasive approaches such as circulating blood biomarkers, to improve early detection.

A notable strength of this study is the inclusion of a relatively large, consecutive, clinically relevant patient population, all of whom had been referred to a lung specialist clinic. The study leveraged a broad set of variables encompassing demographics, medical history, and detailed symptom descriptions, allowing for a nuanced exploration of potential predictors of lung cancer at varying stages. Including patients already referred to a lung specialist adds clinically meaningful value informing patient stratification for diagnostic workup of lung cancer. However, this cohort includes individuals with more pronounced and persistent symptoms, which may limit the generalisability of our findings to primary care settings or the general population, where symptom prevalence and severity may differ. Some considerations related to questionnaire design, such as combining several background factors into a single question, should be noted since they might limit the interpretability. Another limitation is the potential unreliability of recalling past events, for example, past antibiotic use. Lastly, it is important to note that the machine learning analysis was not optimised to produce a single model for clinical use but was instead used to assess the potential of combining background and symptom variables for lung cancer detection. Yet, the hyperparameter tuning and performance estimation within these exploratory machine learning models were performed within the same cross validation procedure, which could result in optimistic performance estimates. Therefore, the generalisability of the models should be evaluated in external cohorts in the future. In addition, calibration assessment indicated that the non-advanced stage models were underfit, highlighting a need to further increase sample size to better explore the more subtle and variable signs of early-stage disease.

In conclusion, this study highlights the diagnostic complexity of lung cancer, particularly in its early stages, where symptom overlap with benign conditions with similar clinical presentation poses a challenge to accurate detection. While the predictive models showed a moderate ability to distinguish between no cancer and lung cancer at different stages, the models require further optimisation and validation in additional cohorts. Demographic and lifestyle factors, most notably age, smoking status, and living situation, remain crucial predictors, with symptoms such as pain, appetite loss, weight reduction, and respiratory problems providing important indicators for referral for lung investigation. Future efforts should focus on integrating clinical, demographic, and biological data along with patient-reported symptoms to enhance the precision of lung cancer prediction models.

## Supplementary Information

Below is the link to the electronic supplementary material.

Supplementary Material 1

## Data Availability

The data supporting the findings of this study are not openly available due to sensitivity considerations. However, they can be obtained from the corresponding author upon reasonable request. All data are stored in controlled-access repositories at Karolinska Institutet.

## Code availability

The code used for the analyses in this study is publicly available on GitHub at: https://github.com/larseriksson1group/PEX-LC-stage-symptoms^23^.
