# Supplementary material for: Predicting lung cancer stage at diagnosis based on self-reported symptoms and background factors using machine learning models
Source: Sci Rep. 2026 Apr 8;16:11866. doi: 10.1038/s41598-026-46710-8 (PMC13065791; doi:10.1038/s41598-026-46710-8)
Supplement: Supplementary file 1 — Supplementary Material 1 [file 41598_2026_46710_MOESM1_ESM.pdf]

Supplementary Materials for

Predicting lung cancer stage at diagnosis based on self-reported symptoms and background factors using machine learning models

Authors: Tina Gustavell [1, 2], Noora Sissala [3], Maria Pernemalm [3], Haris Babacic [3], Lars E Eriksson [1, 4]

Affiliations:

[1] Department of Neurobiology, Care Sciences and Society, Karolinska Institutet, Stockholm, Sweden.

[2] Department of Upper Abdominal Diseases, Theme Cancer, Karolinska University Hospital, Stockholm, Sweden.

[3] Department of Oncology-Pathology, Karolinska Institutet, Science for Life Laboratory, Stockholm, Sweden.

[4] School of Health and Medical Sciences, City St George's, University of London, London, United Kingdom.

Corresponding author: Tina Gustavell, tina.gustavell@ki.se

This document contains

- Supplementary Figures 1-6
- Supplementary Tables 1-4

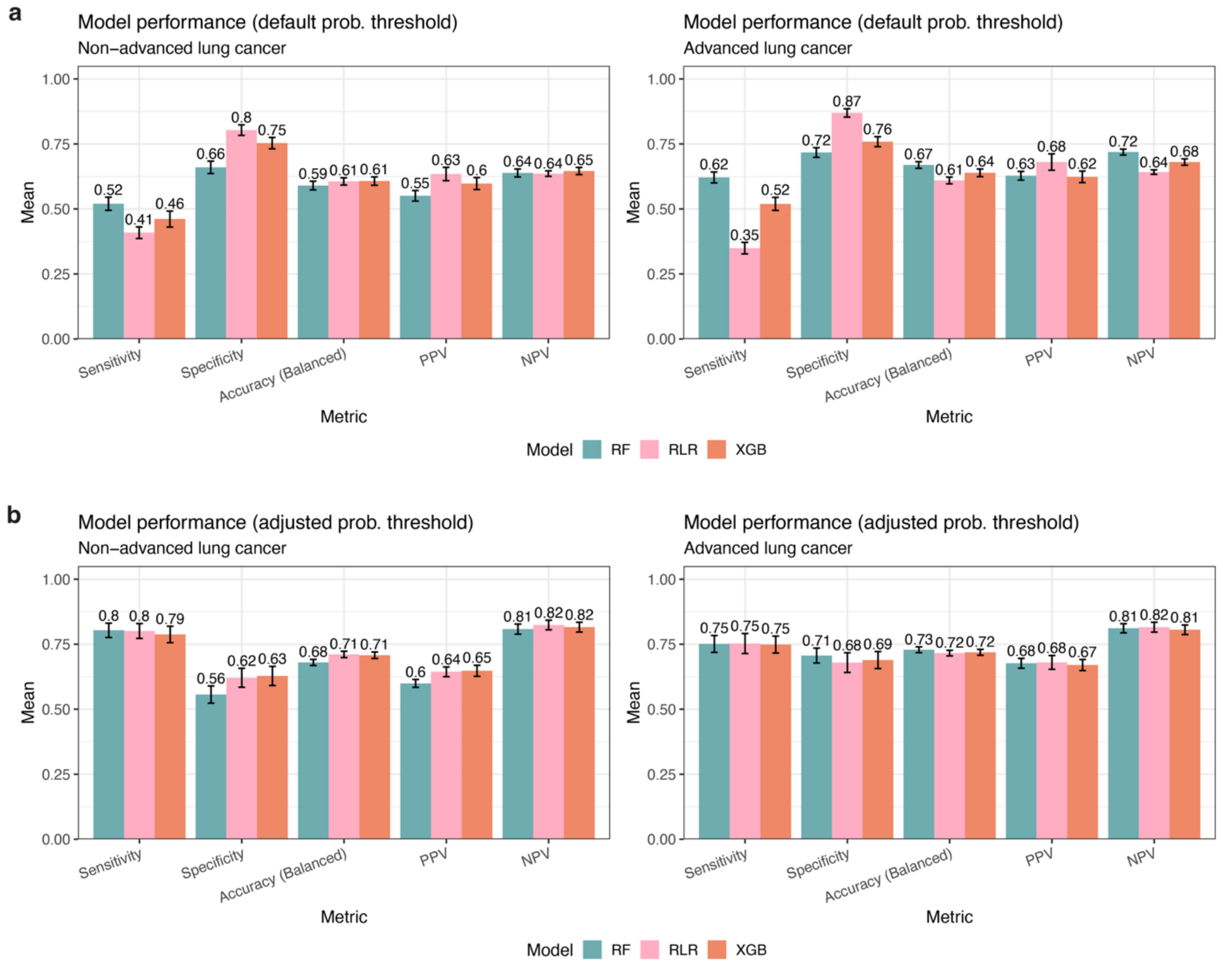

Supplementary Figure 1. Cross validated model performance metrics. a Cross validated model performance metrics for non-advanced (left) and advanced (right) stage models, calculated at the default predicted probability threshold of 0.5 for classifying a sample as lung cancer. The mean over cross validation folds (n = 100) is annotated above each bar. Error bars indicate 95% confidence intervals (CI). **b** Same as (a) but calculated at adjusted probability thresholds, selected based on highest Youden's index, in each cross-validation fold. The selected threshold values are summarized in Supplementary Table 3.

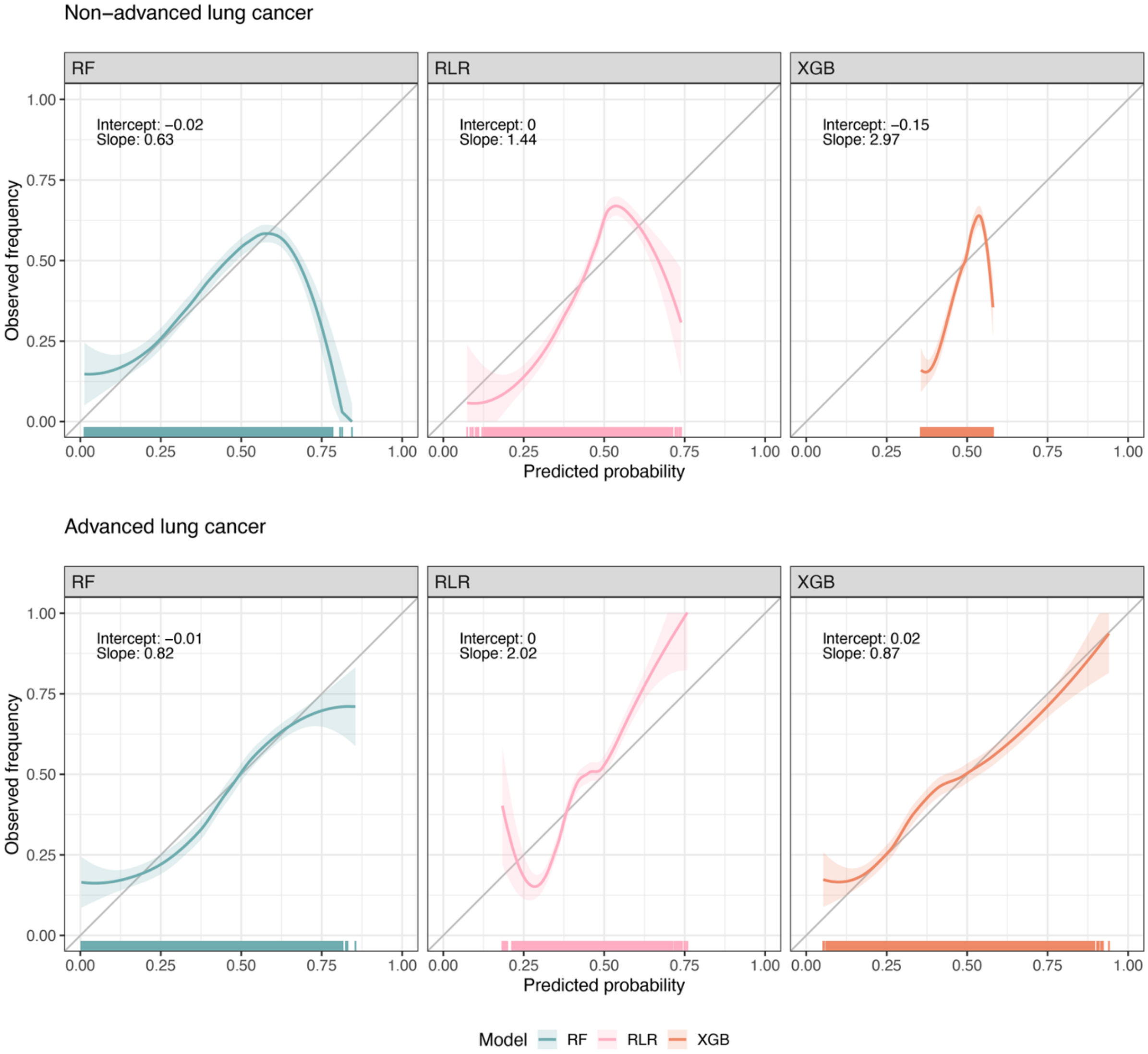

Supplementary Figure 2. Model calibration curves. Comparison of predicted lung cancer probabilities to observed lung cancer frequencies in the hold-out validation sets from all cross-validation iterations. Calibration curves were generated using LOESS-smoothing. Calibration slopes and intercepts, based on a logistic calibration curve, are shown on each plot. Abbreviations: regularised logistic regression (RLR), random forest (RF), extreme gradient boosting (XGB).

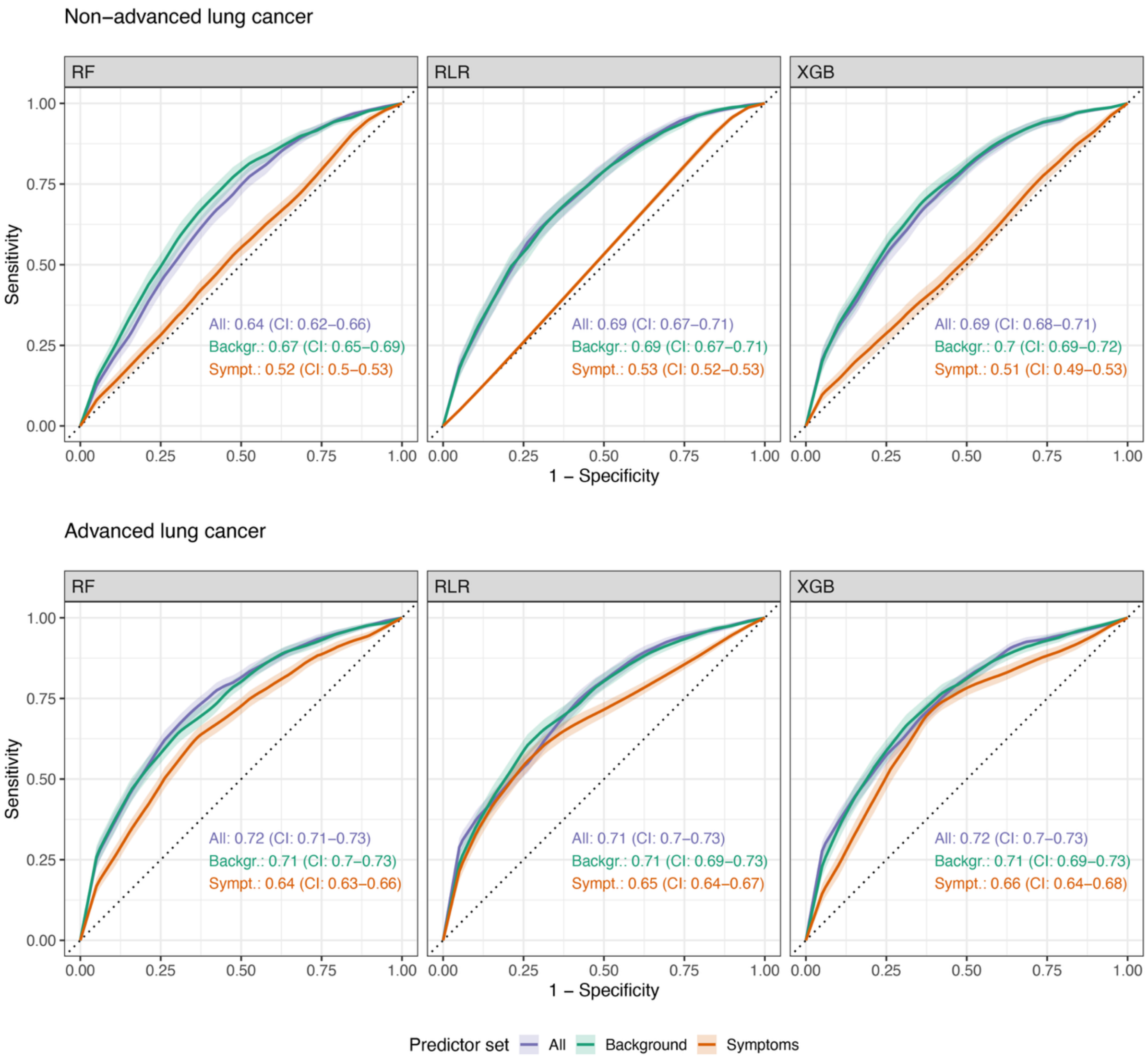

Supplementary Figure 3. Machine learning model performance by predictor set. Receiver operating characteristic (ROC) curves and area under the curve (AUC) for regularised logistic regression (RLR), random forest (RF), and extreme gradient boosting (XGB) models trained with all available predictors (‘All’), background factors only (‘Background’/’Backgr.’), or symptoms only (‘Symptoms’/’Sympt’). AUC is reported as mean over cross validation folds and repeats, with 95% confidence intervals in brackets.

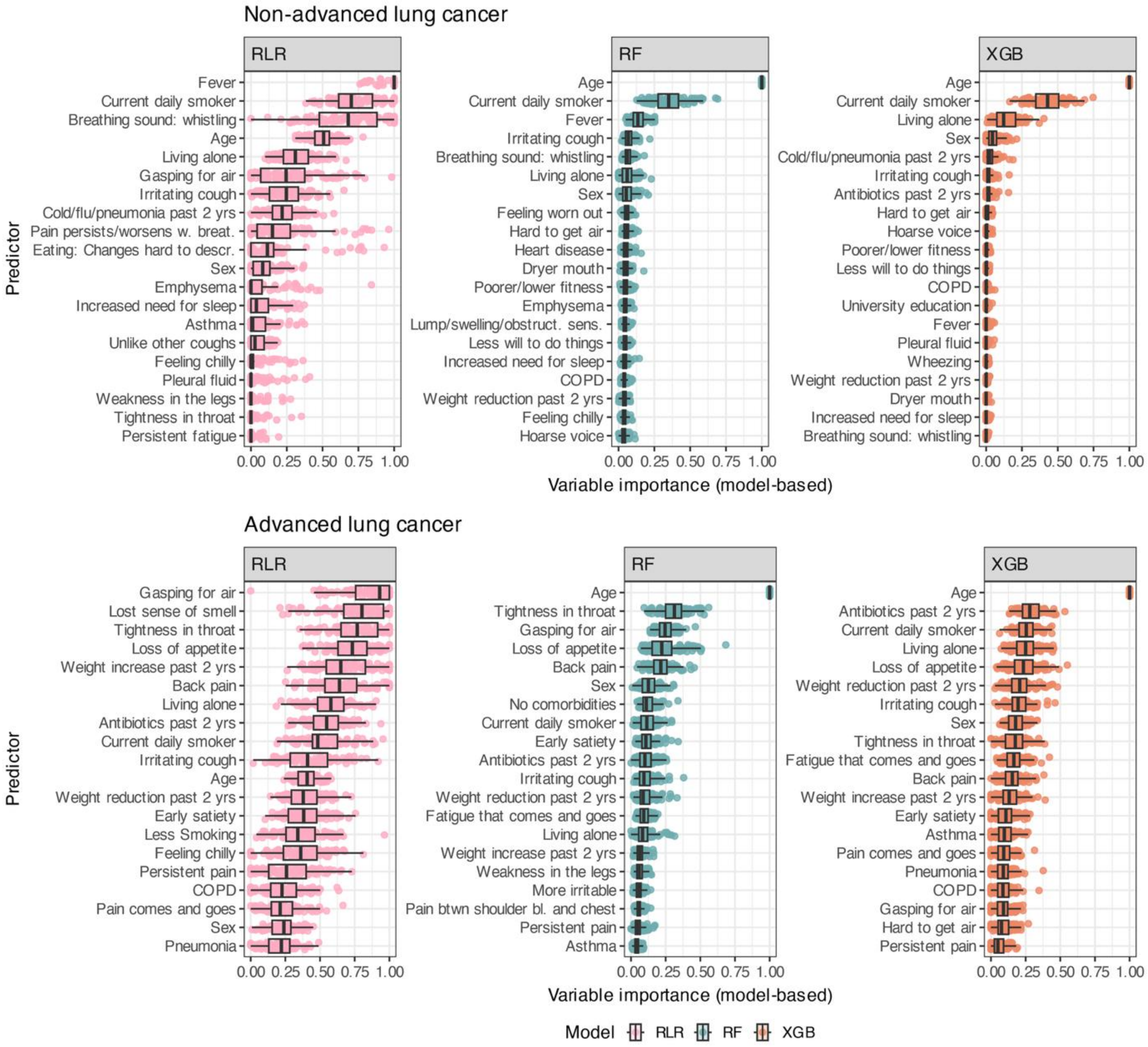

Supplementary Figure 4. Distribution of model-based variable importance estimates across folds. Model estimates of variable importance were extracted from the trained models and scaled, per algorithm, into a range of 0-1. Abbreviations: regularised logistic regression (RLR), random forest (RF), extreme gradient boosting (XGB).

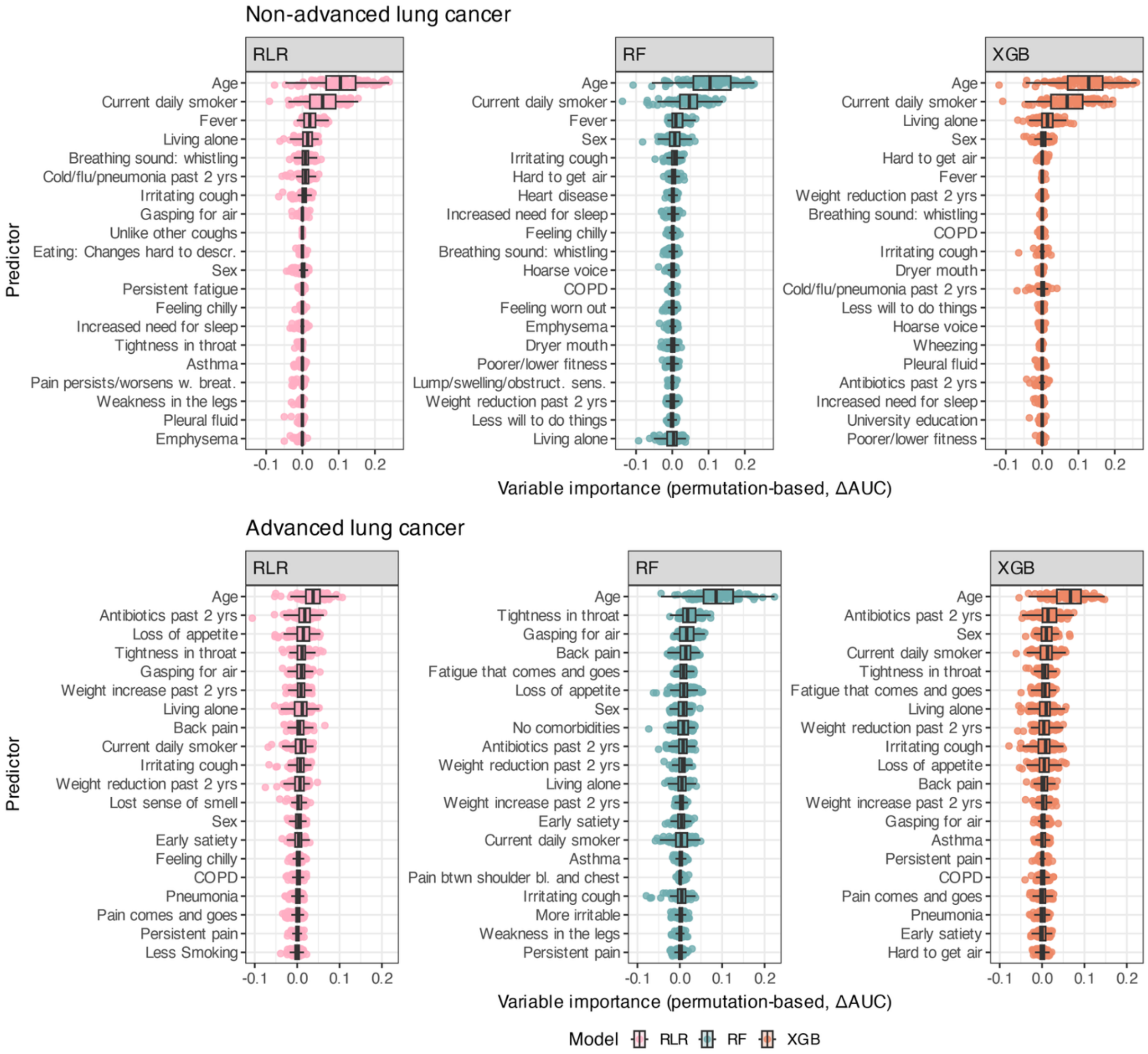

Supplementary Figure 5. Distribution of permutation-based variable importance estimates across folds. Variable importance was calculated through a permutation approach measuring the change in model performance, in terms of area under the curve (AUC), upon permutation of the predictor's values. Abbreviations: regularised logistic regression (RLR), random forest (RF), extreme gradient boosting (XGB).

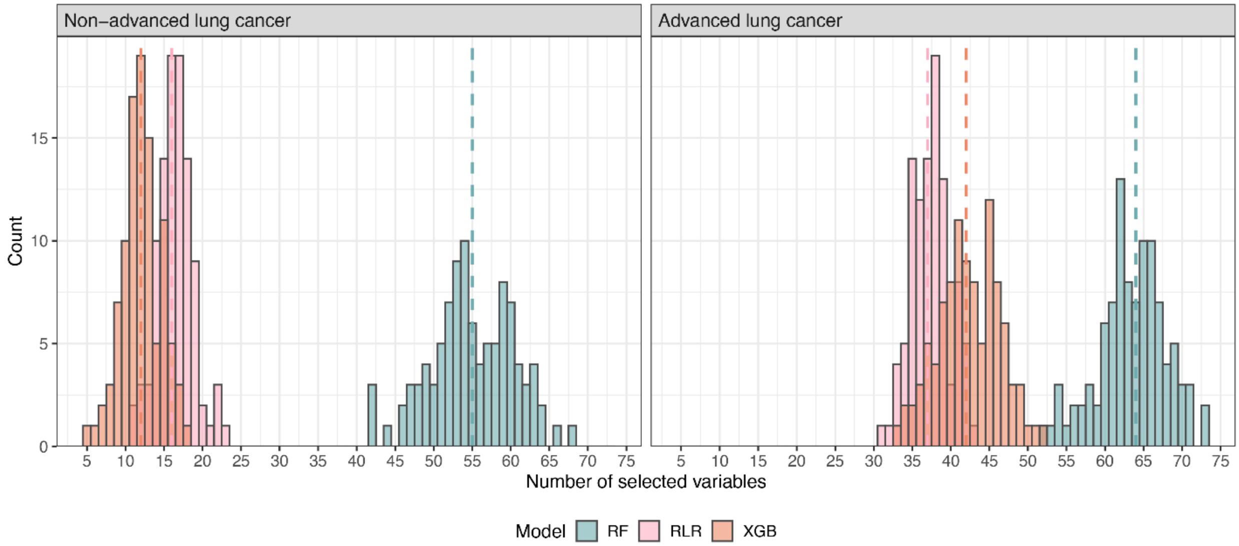

Supplementary Figure 6. Number of selected variables across models and cross-validation folds. Medians are indicated with dashed lines. Selected variables were defined as variables with a model-based variable importance > 0. Abbreviations: regularised logistic regression (RLR), random forest (RF), extreme gradient boosting (XGB).

Supplementary Table 1. Descriptions and comparisons of symptoms reported by patients without cancer (n=192), non-advanced stage lung cancer (n=150) and advanced stage lung cancer (n=144).

	Total (n=486)	No Cancer (n=192)	Non-advanced stage (n=150)					Advanced stage (n=144)				
Symptom / sensation	n (%)	n (%)	n (%)	OR[a]	95% CI[a]		P[a]	n (%)	OR[b]	95% CI[b]		P[b]
Any breathing difficulty	246 (50.62)	87 (45.31)	74 (49.33)	1.18	0.77	1.80	0.460	85 (59.03)	1.74	1.13	2.70	**0.013**
Hard to get air	81 (16.67)	29 (15.1)	15 (10)	0.62	0.31	1.20	0.165	37 (25.69)	1.94	1.13	3.37	**0.017**
Tightness of the chest	40 (8.23)	12 (6.25)	13 (8.67)	1.42	0.63	3.26	0.396	15 (10.42)	1.74	0.79	3.92	0.169
Hard to breathe deeply	79 (16.26)	31 (16.15)	20 (13.33)	0.80	0.43	1.46	0.469	28 (19.44)	1.25	0.71	2.20	0.432
Laboured breathing	116 (23.87)	43 (22.4)	32 (21.33)	0.94	0.56	1.57	0.814	41 (28.47)	1.38	0.84	2.27	0.204
Wheezing	139 (28.60)	49 (25.52)	41 (27.33)	1.10	0.67	1.78	0.706	49 (34.03)	1.51	0.94	2.42	0.090
Hard to catch breath	28 (5.76)	8 (4.17)	6 (4)	0.96	0.31	2.82	0.939	14 (9.72)	2.48	1.03	6.36	**0.048**
Gasping for air	22 (4.53)	3 (1.56)	8 (5.33)	3.55	1.01	16.42	0.065	11 (7.64)	5.21	1.59	23.36	**0.013**
Feeling of choking	19 (3.91)	6 (3.12)	9 (6)	1.98	0.70	6.02	0.205	4 (2.78)	0.89	0.22	3.16	0.853
Sensation of discomfort	44 (9.05)	15 (7.81)	14 (9.33)	1.21	0.56	2.61	0.617	15 (10.42)	1.37	0.64	2.93	0.409
Panic sensation	24 (4.94)	7 (3.65)	8 (5.33)	1.49	0.52	4.34	0.452	9 (6.25)	1.76	0.64	5.04	0.273
Stabbing sensation when taking deep breaths	16 (3.29)	7 (3.65)	3 (2)	0.54	0.11	1.98	0.377	6 (4.17)	1.15	0.36	3.53	0.807
Tightness in throat	39 (8.02)	8 (4.17)	12 (8)	2.00	0.81	5.23	0.140	19 (13.19)	3.50	1.53	8.71	**0.004**
Feels like a lump inside the chest	13 (2.67)	5 (2.6)	3 (2)	0.76	0.15	3.16	0.715	5 (3.47)	1.35	0.37	4.92	0.644
Soreness in the chest	9 (1.85)	3 (1.56)	3 (2)	1.29	0.24	7.03	0.760	3 (2.08)	1.34	0.24	7.34	0.722
Uneasiness hard to describe	41 (8.44)	18 (9.38)	10 (6.67)	0.69	0.30	1.52	0.367	13 (9.03)	0.96	0.45	2.02	0.913
Any breathing sound	110 (22.63)	39 (20.31)	30 (20)	0.98	0.57	1.67	0.943	41 (28.47)	1.56	0.94	2.59	0.083
Breathing sound: squeaky, as if through a pipe	52 (10.70)	21 (10.94)	12 (8)	0.71	0.33	1.47	0.363	19 (13.19)	1.24	0.63	2.40	0.528
Breathing sound: rattly/wheezing	47 (9.67)	15 (7.81)	10 (6.67)	0.84	0.36	1.91	0.687	22 (15.28)	2.13	1.07	4.34	**0.033**
Breathing sound: whistling	24 (4.94)	4 (2.08)	12 (8)	4.09	1.39	14.86	**0.017**	8 (5.56)	2.76	0.85	10.53	0.102
Breathing sound: jarred, raspy	4 (0.82)	1 (0.52)	1 (0.67)	1.28	0.05	32.60	0.861	2 (1.39)	2.69	0.26	58.22	0.421
Breathing sound: bubbly, gurgly	17 (3.50)	7 (3.65)	2 (1.33)	0.36	0.05	1.50	0.203	8 (5.56)	1.55	0.55	4.53	0.405
Breathing sound: hissing	21 (4.32)	7 (3.65)	7 (4.67)	1.29	0.43	3.86	0.637	7 (4.86)	1.35	0.45	4.03	0.582
Any symptoms of cough	232 (47.74)	88 (45.83)	69 (46)	1.01	0.66	1.55	0.976	75 (52.08)	1.28	0.83	1.98	0.257
Mucus cough	125 (25.72)	47 (24.48)	38 (25.33)	1.05	0.64	1.71	0.856	40 (27.78)	1.19	0.72	1.94	0.495

Dry cough	99 (20.37)	43 (22.4)	25 (16.67)	0.69	0.40	1.19	0.189	31 (21.53)	0.95	0.56	1.60	0.849
Barking cough	16 (3.29)	6 (3.12)	4 (2.67)	0.85	0.21	3.03	0.803	6 (4.17)	1.35	0.41	4.39	0.612
Hacking cough	10 (2.06)	5 (2.6)	2 (1.33)	0.51	0.07	2.38	0.419	3 (2.08)	0.80	0.16	3.30	0.757
Wheezing cough	5 (1.03)	3 (1.56)	0 (0)	0.00		8.89×10^{40}	0.985	2 (1.39)	0.89	0.12	5.42	0.897
Irritating cough	74 (15.23)	20 (10.42)	26 (17.33)	1.80	0.97	3.41	0.065	28 (19.44)	2.08	1.12	3.90	**0.021**
Loss of breath due to coughing	23 (4.73)	9 (4.69)	7 (4.67)	1.00	0.35	2.74	0.993	7 (4.86)	1.04	0.36	2.86	0.941
Coughing fits	68 (13.99)	29 (15.1)	18 (12)	0.77	0.40	1.43	0.409	21 (14.58)	0.96	0.52	1.76	0.894
Difficulty suppressing the cough	19 (3.91)	8 (4.17)	6 (4)	0.96	0.31	2.82	0.939	5 (3.47)	0.83	0.25	2.53	0.744
Slight cough	29 (5.97)	12 (6.25)	6 (4)	0.63	0.21	1.65	0.359	11 (7.64)	1.24	0.52	2.91	0.618
Need to clear throat	68 (13.99)	27 (14.06)	22 (14.67)	1.05	0.57	1.93	0.874	19 (13.19)	0.93	0.49	1.74	0.819
Unlike other coughs	5 (1.03)	3 (1.56)	0 (0)	0.00		8.89×10^{40}	0.985	2 (1.39)	0.89	0.12	5.42	0.897
Increased amount of phlegm/expectorates	57 (11.73)	22 (11.46)	14 (9.33)	0.80	0.38	1.60	0.526	21 (14.58)	1.32	0.69	2.51	0.397
White, yellow, or green phlegm/expectorated	45 (9.26)	20 (10.42)	15 (10)	0.96	0.46	1.93	0.900	10 (6.94)	0.64	0.28	1.39	0.272
Blood-mixed phlegm/expectorates	28 (5.76)	8 (4.17)	10 (6.67)	1.64	0.63	4.41	0.308	10 (6.94)	1.72	0.66	4.61	0.268
Any change in phlegm texture	46 (9.47)	25 (13.02)	10 (6.67)	0.48	0.21	1.00	0.059	11 (7.64)	0.55	0.25	1.14	0.118
Lumps/pieces in sputum	10 (2.06)	7 (3.65)	2 (1.33)	0.36	0.05	1.50	0.203	1 (0.69)	0.18	0.01	1.05	0.116
Thick consistency	22 (4.53)	13 (6.77)	5 (3.33)	0.47	0.15	1.29	0.166	4 (2.78)	0.39	0.11	1.14	0.109
Thin mucus	8 (1.65)	5 (2.6)	2 (1.33)	0.51	0.07	2.38	0.419	1 (0.69)	0.26	0.01	1.64	0.223
Thick mucus	23 (4.73)	11 (5.73)	5 (3.33)	0.57	0.18	1.60	0.304	7 (4.86)	0.84	0.30	2.19	0.727
Other consistency changes	4 (0.82)	3 (1.56)	0 (0)	0.00		8.89×10^{40}	0.985	1 (0.69)	0.44	0.02	3.48	0.480
Any pain	211 (43.42)	70 (36.5)	63 (42.0)	1.26	0.81	1.96	0.297	78 (54.17)	2.06	1.33	3.21	**0.001**
Persistent pain	45 (9.26)	13 (6.77)	10 (6.67)	0.98	0.41	2.30	0.970	22 (15.28)	2.48	1.22	5.24	**0.014**
Pain comes and goes	93 (19.14)	31 (16.15)	22 (14.67)	0.89	0.49	1.61	0.708	40 (27.78)	2.00	1.18	3.41	**0.011**
Pain persists/worsens when breathing	16 (3.29)	9 (4.69)	1 (0.67)	0.14	0.01	0.74	0.060	6 (4.17)	0.88	0.29	2.51	0.819
Pain reduces/improves when changing body position	15 (3.09)	5 (2.6)	2 (1.33)	0.51	0.07	2.38	0.419	8 (5.56)	2.20	0.72	7.42	0.175
Pain persists/worsens when changing body position	15 (3.09)	3 (1.56)	4 (2.67)	1.73	0.37	8.88	0.479	8 (5.56)	3.71	1.05	17.15	0.056
Pressure sensation	50 (10.29)	18 (9.38)	16 (10.67)	1.15	0.56	2.35	0.692	16 (11.11)	1.21	0.59	2.46	0.602

Lump/swelling or obstruction sensation	26 (5.35)	11 (5.73)	9 (6)	1.05	0.41	2.61	0.916	6 (4.17)	0.72	0.24	1.93	0.520
Heartburn	28 (5.76)	10 (5.21)	6 (4)	0.76	0.25	2.09	0.601	12 (8.33)	1.65	0.69	4.03	0.256
Feeling of uneasiness that is difficult to describe	42 (8.64)	15 (7.81)	13 (8.67)	1.12	0.51	2.44	0.775	14 (9.72)	1.27	0.59	2.74	0.538
Pain in throat	12 (2.47)	49 (25.52)	39 (26)	1.03	0.63	1.67	0.920	5 (3.47)	1.69	0.44	6.94	0.440
Pain in shoulder blade	18 (3.70)	4 (2.08)	3 (2)	0.96	0.19	4.42	0.957	6 (4.17)	1.00	0.32	2.94	>0.999
Pain in shoulders	21 (4.32)	8 (4.17)	4 (2.67)	0.63	0.17	2.04	0.458	9 (6.25)	1.76	0.64	5.04	0.273
Pain in neck	19 (3.91)	7 (3.65)	5 (3.33)	0.91	0.26	2.91	0.876	7 (4.86)	2.40	0.71	9.32	0.169
Pain in chest	58 (11.93)	4 (2.08)	8 (5.33)	2.65	0.82	10.08	0.118	20 (13.89)	0.99	0.52	1.83	0.964
Pain radiating between shoulder blades and chest	17 (3.50)	6 (3.12)	4 (2.67)	0.85	0.21	3.03	0.803	7 (4.86)	1.58	0.52	5.02	0.418
Headache	13 (2.67)	5 (2.6)	4 (2.67)	1.02	0.25	3.94	0.971	4 (2.78)	1.07	0.26	4.11	0.922
Back pain	52 (10.70)	13 (6.77)	13 (8.67)	1.31	0.58	2.93	0.513	26 (18.06)	3.03	1.52	6.32	**0.002**
Pain in whole body	8 (1.65)	3 (1.56)	3 (2)	1.29	0.24	7.03	0.760	2 (1.39)	0.89	0.12	5.42	0.897
Pain that shifts location	7 (1.44)	4 (2.08)	1 (0.67)	0.32	0.02	2.16	0.304	2 (1.39)	0.66	0.09	3.44	0.637
Any symptoms of fatigue	246 (50.62)	105 (54.69)	82 (54.67)	1.00	0.65	1.54	0.997	99 (68.75)	1.82	1.16	2.88	**0.009**
Less energy to do things	151 (31.07)	55 (28.65)	42 (28)	0.97	0.60	1.55	0.895	61 (42.36)	1.83	1.16	2.89	**0.009**
Less will to do things	96 (19.75)	31 (16.15)	35 (23.33)	1.58	0.92	2.72	0.096	33 (22.92)	1.54	0.89	2.68	0.119
Have become weaker	103 (21.19)	44 (22.92)	32 (21.33)	0.91	0.54	1.52	0.727	45 (31.25)	1.53	0.94	2.49	0.088
Weakness in the legs	37 (7.61)	9 (4.69)	14 (9.33)	2.09	0.89	5.16	0.095	15 (10.42)	2.36	1.02	5.78	**0.049**
Difficulty staying awake	53 (10.91)	10 (5.21)	13 (8.67)	1.73	0.74	4.16	0.210	13 (9.03)	1.81	0.77	4.35	0.175
Not feeling well rested	94 (19.34)	41 (21.35)	30 (20)	0.92	0.54	1.56	0.759	32 (22.22)	1.05	0.62	1.77	0.849
Increased need for sleep	94 (19.34)	36 (18.75)	21 (14)	0.71	0.39	1.26	0.244	38 (26.39)	1.55	0.92	2.62	0.096
Feeling worn out	76 (15.64)	32 (16.67)	20 (13.33)	0.77	0.41	1.40	0.395	27 (18.75)	1.15	0.65	2.03	0.620
Feeling abnormally/unhealthily tired	62 (12.76)	22 (11.46)	17 (11.33)	0.99	0.50	1.93	0.971	22 (15.28)	1.39	0.74	2.64	0.306
Feeling out of sorts	73 (15.02)	29 (15.1)	18 (12)	0.77	0.40	1.43	0.409	31 (21.53)	1.54	0.88	2.71	0.130
Persistent fatigue	78 (16.05)	27 (14.06)	13 (8.67)	0.58	0.28	1.15	0.127	24 (16.67)	1.22	0.67	2.22	0.511
Fatigue that comes and goes	57 (11.73)	32 (16.67)	27 (18)	1.10	0.62	1.93	0.746	18 (12.5)	0.71	0.38	1.32	0.290
Feeling of discomfort, uneasiness that is hard to describe	105 (21.60)	11 (5.73)	7 (4.67)	0.81	0.29	2.10	0.663	12 (8.33)	1.50	0.64	3.55	0.352
Any voice changes	171 (35.19)	75 (39.06)	52 (34.67)	0.83	0.53	1.29	0.404	59 (40.97)	1.08	0.70	1.68	0.724
Hoarse voice	99 (20.37)	46 (23.96)	37 (24.67)	1.04	0.63	1.71	0.880	43 (29.86)	1.35	0.83	2.20	0.226
Rougher voice	42 (8.64)	12 (6.25)	10 (6.67)	1.07	0.44	2.55	0.876	19 (13.19)	2.28	1.08	4.99	**0.033**

Weaker voice	27 (5.56)	14 (7.29)	11 (7.33)	1.01	0.43	2.28	0.988	8 (5.56)	0.75	0.29	1.80	0.526
Loss of voice	21 (4.32)	8 (4.17)	5 (3.33)	0.79	0.24	2.43	0.690	6 (4.17)	1.00	0.32	2.94	>0.999
Changes pitch, higher/lower	37 (7.61)	9 (4.69)	7 (4.67)	1.00	0.35	2.74	0.993	9 (6.25)	1.36	0.52	3.56	0.530
Clears throat more often	50 (10.29)	29 (15.1)	19 (12.67)	0.82	0.43	1.51	0.520	19 (13.19)	0.85	0.45	1.58	0.621
Voice changes difficult to describe	60 (12.35)	2 (1.04)	2 (1.33)	1.28	0.15	10.80	0.804	6 (4.17)	4.13	0.94	28.48	0.085
Any eating changes	139 (28.60)	45 (23.44)	42 (28)	1.27	0.78	2.07	0.337	65 (45.14)	2.69	1.69	4.31	**<0.001**
Loss of appetite	97 (19.96)	26 (13.54)	29 (19.33)	1.53	0.86	2.74	0.150	48 (33.33)	3.19	1.88	5.54	**<0.001**
Not enjoying food	56 (11.52)	21 (10.94)	23 (15.33)	1.47	0.78	2.80	0.230	26 (18.06)	1.79	0.97	3.37	0.065
Food/drinks taste different	31 (6.38)	13 (6.77)	9 (6)	0.88	0.35	2.10	0.773	14 (9.72)	1.48	0.67	3.30	0.327
Trouble swallowing	27 (5.56)	6 (3.12)	4 (2.67)	0.85	0.21	3.03	0.803	3 (2.08)	0.66	0.14	2.55	0.561
Early satiety	57 (11.73)	20 (10.42)	18 (12)	1.17	0.59	2.31	0.644	36 (25)	2.87	1.59	5.29	**0.001**
Changes hard to describe	51 (10.49)	5 (2.6)	1 (0.67)	0.25	0.01	1.58	0.209	4 (2.78)	1.07	0.26	4.11	0.922
Any smell changes	55 (11.32)	26 (13.54)	25 (16.67)	1.28	0.70	2.32	0.422	15 (10.42)	0.74	0.37	1.44	0.388
Difficulty sensing smells	25 (5.14)	11 (5.73)	14 (9.33)	1.69	0.75	3.93	0.208	7 (4.86)	0.84	0.30	2.19	0.727
Lost sense of smell	21 (4.32)	11 (5.73)	7 (4.67)	0.81	0.29	2.10	0.663	2 (1.39)	0.23	0.04	0.88	0.060
More sensitive to different smells	16 (3.29)	8 (4.17)	8 (5.33)	1.30	0.47	3.60	0.613	8 (5.56)	1.35	0.49	3.76	0.555
Changes hard to describe	29 (5.97)	2 (1.04)	0 (0)	0.00		2.16×10^{36}	0.982	2 (1.39)	1.34	0.16	11.26	0.772
Any signs or symptoms of fever	100 (20.58)	49 (25.52)	38 (25.33)	0.99	0.60	1.61	0.969	47 (32.64)	1.41	0.88	2.28	0.154
Chills	28 (5.76)	11 (5.73)	4 (2.67)	0.45	0.12	1.35	0.180	5 (3.47)	0.59	0.18	1.67	0.341
Feeling chilly	27 (5.56)	9 (4.69)	12 (8)	1.77	0.73	4.44	0.211	17 (11.81)	2.72	1.20	6.57	**0.019**
Fever	32 (6.58)	15 (7.81)	1 (0.67)	0.08	0.00	0.40	**0.015**	12 (8.33)	1.07	0.48	2.36	0.862
Day sweats; more than usual	28 (5.76)	6 (3.12)	5 (3.33)	1.07	0.30	3.62	0.914	4 (2.78)	0.89	0.22	3.16	0.853
Night sweats	39 (8.02)	21 (10.94)	18 (12)	1.11	0.56	2.17	0.759	18 (12.5)	1.16	0.59	2.27	0.658
Sweating all the time	10 (2.06)	0 (0)	0 (0)					1 (0.69)	$1.05 x 10^6$	0.00		0.979
Cold feet	49 (10.08)	7 (3.65)	9 (6)	1.69	0.61	4.82	0.311	4 (2.78)	0.76	0.19	2.55	0.659
Calf cramp	74 (15.23)	26 (13.54)	29 (19.33)	1.53	0.86	2.74	0.150	19 (13.19)	0.97	0.51	1.82	0.926
Swollen/tender joints	44 (9.05)	20 (10.42)	13 (8.67)	0.82	0.38	1.68	0.587	11 (7.64)	0.71	0.32	1.51	0.386
Nail changes	32 (6.58)	13 (6.77)	12 (8)	1.20	0.52	2.72	0.665	7 (4.86)	0.70	0.26	1.77	0.466
Dryer skin	54 (11.11)	21 (10.94)	17 (11.33)	1.04	0.52	2.05	0.908	16 (11.11)	1.02	0.50	2.02	0.960
Dryer mouth	82 (16.87)	28 (14.58)	28 (18.67)	1.34	0.76	2.39	0.312	26 (18.06)	1.29	0.72	2.32	0.392
Tightness in throat	31 (6.38)	11 (5.73)	7 (4.67)	0.81	0.29	2.10	0.663	13 (9.03)	1.63	0.71	3.83	0.249

Poorer /lower fitness	161 (33.13)	57 (29.69)	50 (33.33)	1.18	0.75	1.88	0.471	54 (37.5)	1.42	0.90	2.25	0.133
Feeling more down	67 (13.79)	26 (13.54)	18 (12)	0.87	0.45	1.65	0.673	23 (15.97)	1.21	0.66	2.23	0.533
More irritable	45 (9.26)	15 (7.81)	15 (10)	1.31	0.62	2.79	0.479	15 (10.42)	1.37	0.64	2.93	0.409
Other mood changes	6 (1.23)	1 (0.52)	4 (2.67)	5.23	0.76	102.95	0.141	1 (0.69)	1.34	0.05	33.97	0.838
Feeling that something is wrong	24 (4.94)	9 (4.69)	5 (3.33)	0.70	0.21	2.08	0.533	10 (6.94)	1.52	0.60	3.92	0.378

[a] Odds ratio (OR), upper and lower values for 95% confidence intervals (CI), and p-value for univariate analysis using logistic regression comparing patients without cancer to patients with non-advanced stage lung cancer.

[b] Odds ratio (OR), upper and lower values for 95% confidence intervals (CI), and p-value for univariate analysis using logistic regression comparing patients without cancer to patients with advanced stage lung cancer.

Supplementary Table 2. Summary of discriminatory performance and model hyperparameters for the best models.

Stage	Model	AUC Mean	AUC SD	AUC 95% CI	Hyperparameters
**Non-advanced stage**	RLR	0.691	0.089	0.674-0.709	alpha: 0.5, lambda: 0.0784759970351461
**Non-advanced stage**	RF	0.642	0.092	0.624-0.660	mtry: 64, splitrule: gini, min.node.size: 30
**Non-advanced stage**	XGB	0.695	0.090	0.677-0.712	eta: 0.01, max_depth: 3, gamma: 5, colsample_bytree: 0.9, min_child_weight: 3, subsample: 0.8, nrounds: 50
**Advanced stage**	RLR	0.711	0.078	0.696-0.727	alpha: 0.1, lambda: 0.335981828628378
**Advanced stage**	RF	0.72	0.072	0.706-0.734	mtry: 129, splitrule: gini, min.node.size = 30
**Advanced stage**	XGB	0.717	0.078	0.701-0.732	eta: 0.1, max_depth: 6, gamma: 5, colsample_bytree: 0.6, min_child_weight: 1, subsample: 0.5, nrounds: 200

AUC = area under the curve. SD = standard deviation. RLR = regularized logistic regression. RF = random forest. XGB = extreme gradient boosting. CI = confidence interval.

Supplementary Table 3. Summary of cross validated performance metrics for lung cancer prediction models. Values represent the mean and 95% confidence intervals (CI) across all folds (n = 100) for balanced accuracy, sensitivity, specificity, negative predictive value (NPV), and positive predictive value (PPV). Metrics were calculated from predictions made with a default probability cutoff of 0.5 for lung cancer classification, and adjusted probability cutoffs determined based on Youden's index in each fold.

	**Non-advanced stage models**			**Advanced stage models**		
	RLR	RF	XGB	RLR	RF	XGB
Measures	Mean (95% CI)	Mean (95% CI)	Mean (95% CI)	Mean (95% CI)	Mean (95% CI)	Mean (95% CI)
Default probability cutoff						
Balanced accuracy	0.61 (0.59-0.62)	0.59 (0.57-0.61)	0.61 (0.59-0.62)	0.61 (0.60-0.62)	0.67 (0.66-0.68)	0.64 (0.62-0.65)
Sensitivity	0.41 (0.39-0.43)	0.52 (0.49-0.55)	0.46 (0.43-0.49)	0.35 (0.33-0.37)	0.62 (0.60-0.64)	0.52 (0.49-0.54)
Specificity	0.80 (0.78-0.82)	0.66 (0.64-0.68)	0.75 (0.73-0.77)	0.87 (0.85-0.89)	0.72 (0.70-0.74)	0.76 (0.74-0.78)
NPV	0.64 0.63-0.65)	0.64 (0.62-0.65)	0.65 (0.63-0.66)	0.64 (0.63-0.65)	0.72 (0.71-0.73)	0.68 (0.67-0.69)
PPV	0.63 (0.61-0.66)	0.55 (0.53-0.57)	0.60 (0.57-0.62)	0.68 (0.65-0.71)	0.63 (0.61-0.64)	0.62 (0.60-0.65)
Adjusted probability cutoff						
Balanced accuracy	0.71 (0.70-0.72)	0.68 (0.67-0.69)	0.71 (0.70-0.72)	0.72 (0.71-0.73)	0.73 (0.72-0.74)	0.72 (0.71-0.73)
Sensitivity	0.80 (0.77-0.83)	0.80 (0.78-0.83)	0.79 (0.76-0.82)	0.75 (0.71-0.79)	0.75 (0.72-0.78)	0.75 (0.72-0.78)
Specificity	0.62 (0.58-0.66)	0.56 (0.52-0.59)	0.63 (0.59-0.66)	0.68 (0.64-0.72)	0.71 (0.68-0.73)	0.69 (0.66-0.72)
NPV	0.82 (0.81-0.84)	0.81 (0.79-0.83)	0.82 (0.80-0.83)	0.82 (0.80-0.83)	0.81 (0.79-0.83)	0.81 (0.79-0.82)
PPV	0.64 (0.63-0.66)	0.60 (0.58-0.61)	0.65 (0.63-0.67)	0.68 (0.65-0.71)	0.68 (0.66-0.70)	0.67 (0.65-0.69)

RLR = regularised logistic regression; RF = random forest; XGB = extreme gradient boosting (XGBoost), CI = confidence interval.

Supplementary Table 4. Summary statistics across cross validation folds for adjusted probability thresholds, selected by highest Youden's index in each fold.

Stage	Model	Metric	n	Mean	SD	Median	Min	Max	SE	95% CI	
**Advanced stage**	RF	threshold	100	0.402	0.091	0.401	0.116	0.6	0.009	0.384	0.42
**Advanced stage**	RLR	threshold	100	0.421	0.049	0.43	0.262	0.562	0.005	0.411	0.43
**Advanced stage**	XGB	threshold	100	0.473	0.03	0.474	0.365	0.55	0.003	0.467	0.479
**Non-advanced stage**	RF	threshold	100	0.454	0.087	0.456	0.211	0.649	0.009	0.437	0.471
**Non-advanced stage**	RLR	threshold	100	0.421	0.053	0.409	0.334	0.568	0.005	0.41	0.431
**Non-advanced stage**	XGB	threshold	100	0.41	0.105	0.406	0.189	0.677	0.01	0.389	0.431

RLR = regularized logistic regression. RF = random forest. XGB = extreme gradient boosting. SD = standard deviation. CI = confidence interval.
